# Sacral Emphysematous Osteomyelitis Caused by* Escherichia coli* after Arthroscopy of the Knee

**DOI:** 10.1155/2016/1961287

**Published:** 2016-08-29

**Authors:** Mirko Velickovic, Thomas Hockertz

**Affiliations:** Department of Orthopedic Surgery and Traumatology, Städtisches Klinikum Wolfenbüttel, Alter Weg 80, 38302 Wolfenbüttel, Germany

## Abstract

Emphysematous osteomyelitis is a rare but serious condition which is often associated with a fatal outcome. The typical appearances of emphysematous osteomyelitis are clusters of small gas bubbles within the medullary cavity. We report a case of a 62-year-old male who presented with emphysematous osteomyelitis due to hematogenous spread of* Escherichia coli* from the knee after arthroscopy.

## 1. Case Report

**Figure 1:**
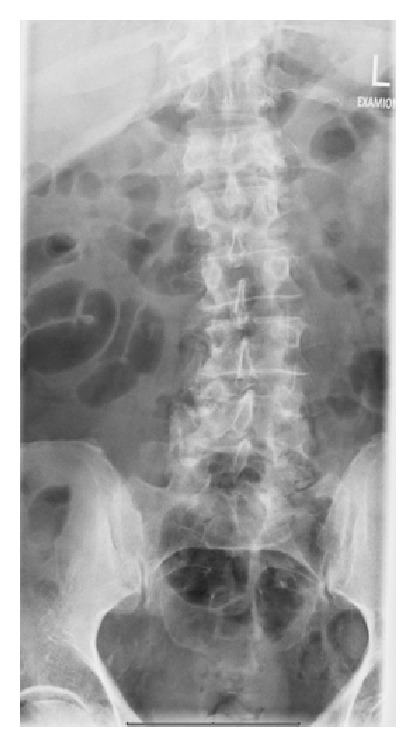
Lumbar spine radiographs on admission showed degenerative changes.

A 62-year-old man presented to our emergency center with severe lower back pain. His complaint started a month ago and the pain in the lower spine was now increasing. Oral painkillers did not produce significant pain relief. The patient denied any history of trauma. On admission to hospital, the patient suffered from lower back pain radiating to the left buttock but not to the leg. There was no numbness or any neurologic deficit. The rest of the physical examination was without any pathologic findings. The patient was febrile (38.4°), blood pressure was 142/88 mmHg, heart rate was 103/minute, and SaO_2_ was 98%. Routine laboratory investigations revealed raised CRP 132,7 mg/L, and the leukocytes were normal. Glucose blood levels were slightly raised up to 206 mg/dL. Lumbar spine radiographs showed degenerative changes but no fracture (Figure 1). The medical history revealed that the patient had an arthroscopy of the right knee two months ago with total synovectomy, chondroshaving, and partial meniscectomy of the medial meniscus as well as lateral release. The physical examination of the knee was unspecific except for slight effusion in the knee with very few symptoms. There was no redness, swelling, atrophy, or any deformity. The scars were healed. The patient walked normally without any limping and with no gait disturbances. There was no need for imaging the knee in the emergency center. The patient was admitted to the ward. Control laboratory examination on the next day showed a marked increase of CRP 164,7 (mg/L—Normal < 5 mg/L) as well as leukocytes (16,2 × 10^3^/*μ*L—Normal 4–11 × 10^3^/*μ*L). Procalcitonin was however markedly increased (1,70 ng/mL—Normal 0,05–0,5 ng/mL), and HBA1C was normal. We started an antibiotic treatment with Amoxiclav (Amoxicillin and Clavulanic Acid) intravenously. The first blood cultures (aerobe and anaerobe) verified the presence of a multisensible* Escherichia coli* strain. Transthoracic and transesophageal echocardiography excluded endocarditis. A sonography of the abdomen was inconspicuous.

**Figure 2:**
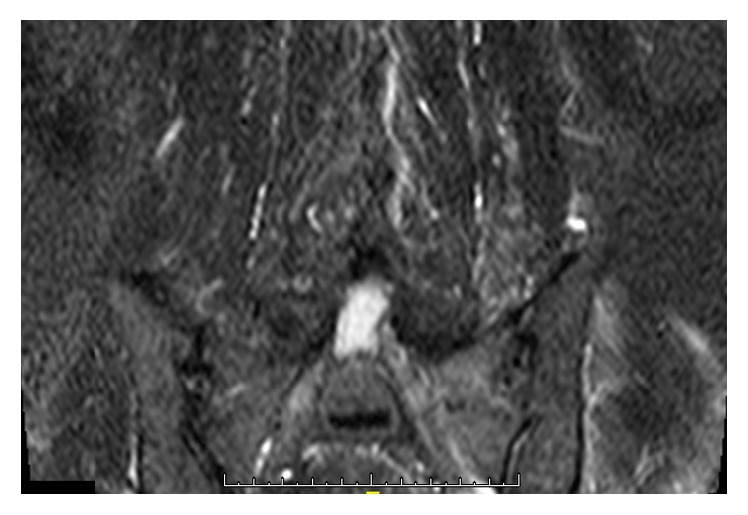
MRI with osteomyelitis of the sacrum as well as free air bubbles in the sacrum.

**Figure 3:**
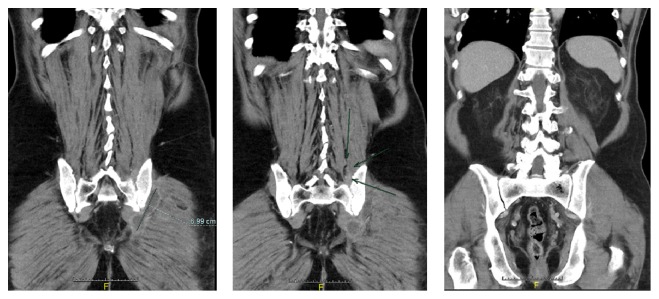
CT scan with multiple free air bubbles in the massa lateralis of the sacrum as well as edema of the iliacus and lumbricales muscles of the left side.

**Figure 4:**
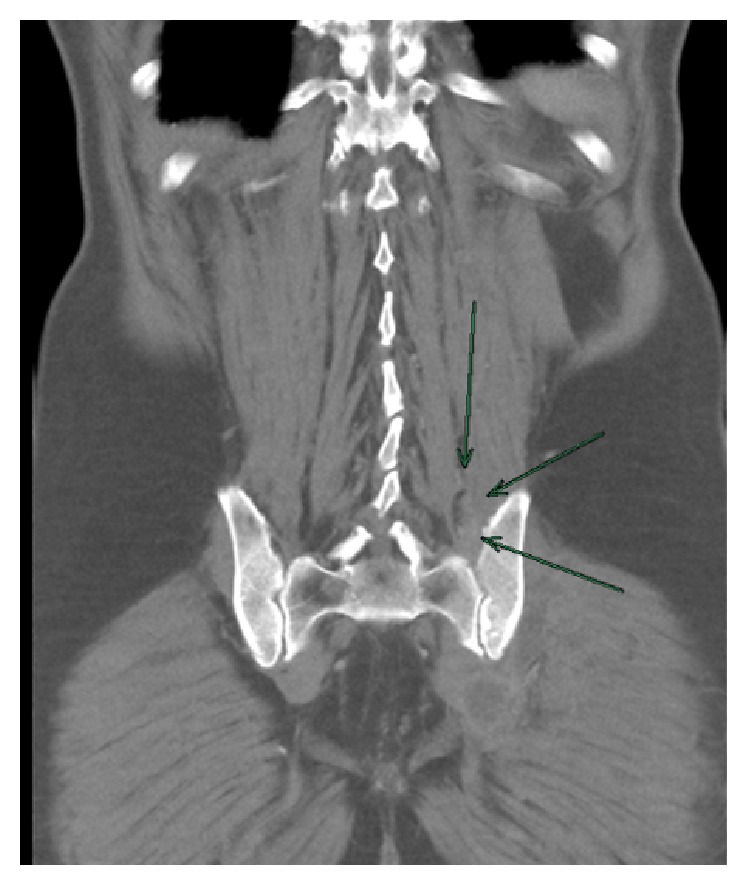
Abscess formation around the piriformis muscle and beginning osteolytic destruction of the ilium bone.

**Figure 5:**
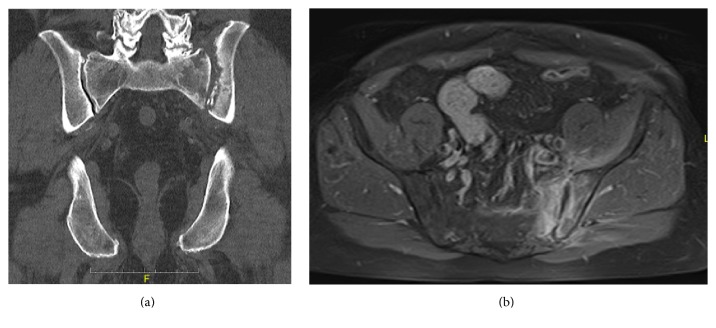
Control CT and MRI of the pelvis 2 months later: osteolytic destruction of the massa lateralis with fluid around the piriformis muscle and the ilium bone.

**Figure 6:**
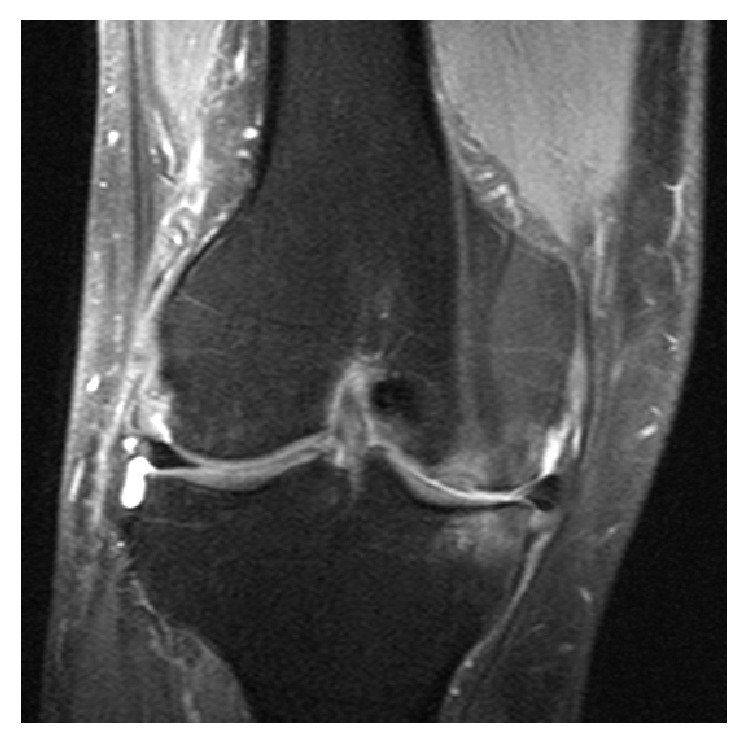
MRI of the knee: inflammatory changes with marked effusion and synovitis as well as subchondral edema at the medial tibia and the medial femur.

Stool analysis excluded the presence of pathologic germs. Due to the strong pain in the lower back, we assumed spondylodiscitis but this was excluded by MRI. But the MRI demonstrated the presence of osteomyelitis of the massa lateralis of the sacrum (Figure 2). The additional CT scan of the abdomen and pelvis showed edema of the iliacus and lumbricales muscles of the left side as well as splenomegaly and nephritis and multiple free air bubbles in the massa lateralis of the sacrum (Figure 3). In the follow-up CT scan of the pelvis 12 days later, there was no free air any more detectible but there was an abscess formation around the piriformis muscle and beginning osteomyelitis of the ilium bone (Figure 4). There were also no more signs of nephritis detectible. Surgery was strongly recommended but the patient refused any kind of surgical interventions including open surgical debridement of the sacrum as well as drainage of the abscess by interventional radiologic methods such as percutaneous catheterization. We continued the high dose antibiotic treatment and performed regular laboratory examinations. The patient clinically improved, and the symptoms markedly declined. There was no fever any more. On discharge after 31 days in hospital, leucocytes were normal and CRP was 17,3 mg/L. There were 4 follow-up examinations in our outpatient clinic in 2 months. As part of the control, we performed a CT scan and an MRI of the pelvis one month after discharge from hospital which showed marked osteolytic destruction of the massa lateralis of the sacrum but there was a decline of the abscess formation along the piriformis muscle (Figures 5(a) and 5(b)). In one of the outpatient follow-up examinations, the patient complained of pain and gait disturbances in the knee so an MRI of the knee was done. This demonstrated extensive effusion of the knee with contrast accumulation compatible with a synovitis as a sign of the inflammatory changes and zones of subchondral edema at the medial tibia and the medial femur (Figure 6). The patient again refused any surgical intervention and did not apply for follow-up examinations. In total, Amoxiclav (Amoxicillin and Clavulanic Acid) was administered intravenously for 3 weeks and orally for 9 weeks.

## 2. X-Rays

See Figures 1, 2, 3, 4, 5, and 6.

## 3. Discussion

Emphysematous osteomyelitis is a rare but serious condition and may result in a fatal outcome. It was first described by Ram and colleagues in 1981 [1]. There are less than 30 cases of emphysematous osteomyelitis reported in English literature. Most cases are associated with immunosuppression due to diabetes mellitus or malignancies [2–4]. However, our patient was negatively tested to have diabetes mellitus. A malignancy was not known previously and there were no signs of malignancy in the CT or MRI imaging so we considered a malignancy as the origin to be unlikely. Nevertheless, additional diagnostic procedures such as oncologic examinations or colonoscopy can be useful especially in cases of a positive family history for malignancies. Blood cultures revealed the presence of* Escherichia coli* which was treated according to the resistogram. Most cases of emphysematous osteomyelitis are monomicrobial and are caused either by an anaerobe or as in our case by a member of the Enterobacteriaceae family although causative organisms include the whole spectrum of aerobic and anaerobic bacteria. Monomicrobial infections are most commonly due to hematogenous spread and polymicrobial infections due to contiguous spread. Nearly every bone can be affected but most cases occur in the vertebrae, sacrum, and lower extremities [2].

We cannot prove that the development of the osteomyelitis of the sacrum was unequivocally based on the arthroscopy of the knee, but we consider hematogenous spread from the knee to be very likely. Another source for the infection could not be found. Cases of spontaneous of emphysematous osteomyelitis are described in literature but in our case the patient underwent surgery of the knee and complained about constant symptoms with slight pain during the in-patient stay. Although the physical examination of the knee was still unremarkable, the MRI of the knee demonstrated signs of inflammatory changes with marked effusion, synovitis, and subchondral edema at the medial tibia and the medial femur. The presence of intraosseous gas is not always pathologic. Degenerative joint diseases as well as vacuum phenomena, ischemic necrosis, malignancies, and cysts may lead to accumulation of gas. However, intraosseous gas together with systematic symptoms such as fever has to be considered as a serious condition and may be caused by gas-forming organisms. Emphysematous osteomyelitis is associated with high mortality (32%); therefore, immediate diagnosis and treatment are crucial for the outcome. Ordinary X-rays showed intraosseous gas so we performed a CT scan not only to confirm the X-ray findings but also to show the extent and exact location of the gas formation. To reveal the presence of an additional abscess or soft tissue process, an MRI is recommended. In most cases, early surgical treatment is essential. In our case, surgery was recommended but the patient refused any surgical intervention. Due to extensive antibiotic therapy, the patient survived. On discharge, there were no signs of fever anymore detectible and the laboratory control examination showed nearly normal values.

## 4. Conclusion 

Emphysematous osteomyelitis is a rare but potentially fatal condition. It should always be considered whenever intraosseous gas is identified on imaging. Most reported cases are secondary to an intra-abdominal infection or surgery or are associated with immunosuppression like in malignancies or diabetes mellitus so taking history is crucial. Diagnostic procedures should include X-rays, CT, and MRI laboratory examinations and exclude or include comorbidities such as diabetes mellitus and malignancies. In general, early and extensive treatment is crucial for an adequate outcome.
